# Growth Performance Analysis of Two Italian Slow-Growing Chicken Breeds: *Bianca di Saluzzo* and *Bionda Piemontese*

**DOI:** 10.3390/ani10060969

**Published:** 2020-06-03

**Authors:** Dominga Soglia, Stefano Sartore, Sandra Maione, Achille Schiavone, Sihem Dabbou, Joana Nery, Luisa Zaniboni, Stefano Marelli, Paola Sacchi, Roberto Rasero

**Affiliations:** 1 Department of Veterinary Science, University of Turin, Largo P. Braccini 2, 10095 Grugliasco, Italy; dominga.soglia@unito.it (D.S.); stefano.sartore@unito.it (S.S.); sandra.maione@unito.it (S.M.); joana.nery@unito.it (J.N.); paola.sacchi@unito.it (P.S.); roberto.rasero@unito.it (R.R.); 2 Center Agriculture Food Environment (C3A), University of Trento, Via E. Mach 1, 38010 San Michele all’Adige, Italy; sihem.dabbou@unitn.it; 3 Research and Innovation Centre, Fondazione Edmund Mach, 38010 San Michele all’Adige, Italy; 4 Department of Veterinary Medicine, University of Milan, via dell’Università 6, 26900 Lodi, Italy; luisa.zaniboni@unimi.it (L.Z.); stefano.marelli@unimi.it (S.M.)

**Keywords:** poultry, Gompertz model, growth, local breeds, *PAX7*, conservation value

## Abstract

**Simple Summary:**

*Bianca di Saluzzo* and *Bionda Piemontese* are two Italian slow-growing chicken breeds of the Piemonte region (Northwest Italy) and are reared mainly for meat. They conserve adaptation to free ranging low input rearing systems and are promising for antibiotic-free farming. We aimed to analyze their growth using a mathematical model and to obtain some advice for improving performance. Polymorphism of the *PAX7* gene was also studied to increase conservation value. The results confirmed that these breeds are late-maturing. Whereas selection would increase inbreeding, a mating scheme to bring inbreeding under control could be the most proper way to increase growth rate and reach commercial maturity earlier.

**Abstract:**

*Bianca di Saluzzo* (BS) and *Bionda Piemontese* (BP) are two Italian chicken breeds, mainly reared for meat production, primarily in antibiotic-free farming. However, technical information on their growth pattern is still missing. At hatching, 150 unsexed chicks of each breed were weighed, labeled, and reared in indoor pens up to 8 w of age. At 8 w of age, the chicks were separated by sex and randomly transferred to growing pens with access to an external paddock (15 birds/pen; 4 pens/sex for each breed). The body weight (BW) was recorded biweekly for each bird, from hatching to 32 w of age. In order to identify an improvement strategy, the objectives of our study were to analyze the growth pattern of these birds using the Gompertz mathematical model and compare results with other chicken breeds. Polymorphism of the *PAX7* gene was also analyzed to test its association with growth traits. Both BS and BP are close to unselected native breeds and, among the Italian local poultry, they are confirmed to be slow-growing birds with an intermediate size between heavy and light chicken breeds. Regarding the *PAX7* gene, two alleles were found, F and G, and showed an association with the actual BW in the BP females from 14 w of age onwards. The G allele always exhibited a more favorable effect than the F allele. In small size poultry population, a delicate balance between preservation of biodiversity and performance improvement should be considered. Consequently, the most proper way could be an approach based on a mating scheme to keep inbreeding under control, increase growth rate, and improve commercial maturity.

## 1. Introduction

Conservation of native breeds is an important component of poultry biodiversity. Local production of meat and eggs needs to be increased, although commercial hybrids have a higher aptitude for growing and laying. Worldwide, industrial systems provide 67% of poultry meat and 50% of egg production; therefore, local poultry populations are given some potential economic profit [1].

The Food and Agriculture Organization of the United Nations (FAO) stated that 55% of the overall local avian breeds are located in Europe and in Caucasian regions [1]. In Italy, 22 breeds have been included in the Registry of Indigenous Poultry and most of them are included in the FAO Domestic Animal Diversity Information System (DAD-IS) database [2,3]. Therefore, the description of morphology, performance and management conditions, as well as collection of genetic and historical information would be necessary to provide contribution to DAD-IS.

In the Piemonte region (Northwest Italy), there are two local chicken breeds, *Bionda Piemontese* (BP) and *Bianca di Saluzzo* (BS) [4]. The BP is characterized by a blond (in Italian “*biondo*”) plumage and a black tail, while the BS is completely white (in Italian “*bianco*”). At present, BP and BS are reared mainly for meat production and are slaughtered at around 180 days (d) of age, although they were formerly considered as dual-purpose breeds. In 2013, the two avian population sizes were 16,000 and 4000 birds for the BP and BS, respectively; thus, the urgent need to promote preservation programs is suggested [5].

Sustainable use of genetic resources in extensive and organic systems is an alternative practice to industrial farming; it is perceived to be more respectful of animal welfare and the environment [6]. BS and BP perform well under these alternative systems and conserve adaptation to low input rearing systems that may positively affect welfare and quality; their products have been officially recognized as traditional and are usually sold as whole carcasses and/or processed meat products [7,8,9,10,11,12,13,14,15]. These properties provide a contribution to conservation value and support the proposal of an *in situ* conservation action. The farmers of BS and BP are included in the consortium for preservation of *Bianca di Saluzzo* and *Bionda Piemontese* breeds and in the project conservation of biodiversity in Italian poultry breeds, which is devoted to the Italian poultry breeds (https://www.pollitaliani.it/en/), and supported by the Italian Ministry of Agricultural, Food, and Forestry Policies [16,17,18].

BS and BP are considered slow growing and late-maturing breeds, nevertheless, this feature is based mainly on anecdotal information and account of farmers; no scientific publication has been produced on this topic so far. Knowledge of growth performance is a fundamental contribution to improvement and conservation of local poultry breeds. Mathematical models, which apply to analysis of growth by fitting estimated weights to actual data, are very useful. These models provide parameters of biological meaning that can be employed in investigation on body composition, protein and mineral deposition, dietary intake, efficiency of nutrient and energy utilization, protein requirements, as well as choice of the best management and breeding strategy [19,20,21,22,23,24]. Gompertz model is one of the equations most frequently used to analyze growth; several versions of the model exist, which were developed in different fields of application, such as growth of plants, animals, bacteria, and tumor cells [25,26,27].

A further tool to describe and evaluate the local poultry breeds is the molecular analysis [4,6]. Investigation on polymorphism of individual candidate genes has more limited prospect than association studies on high-density single nucleotide polymorphism (SNP) arrays, nevertheless, it may be useful to increase the conservation value with a focused approach and to compare different breeds with each other. Paired box 7 (*PAX7*) gene is a marker of precursor cells during myogenesis and it has been proposed as a candidate marker for growth, carcass, and meat quality in a fast-growing chicken line [28].

In the present investigation, the growth performance of BS and BP chickens was assessed. In order to identify a strategy of genetic improvement, the objectives were (1) to analyze the growth from hatching to 32 weeks (w) of age of chicks separated by sex using mathematical models, and (2) to compare the obtained results with the growth pattern of other breeds/lines. In addition (3), the polymorphism of the *PAX7* gene was analyzed to test its association with growth and to increase the conservation value of the two breeds.

## 2. Materials and Methods

### 2.1. Ethical Statement

Qualified co-author veterinarians performed all handling practices aimed at identification, sexing, and weighing of chickens. Blood samples were collected at once during routine health controls by the public veterinary service. No action involving pain or suffering was practiced. The experimental protocol was approved by the Bioethical Committee of the University of Turin (prot. n. 451944).

### 2.2. Management, Identification, Sexing, and Weighing of Chickens

All chicks were purchased from the same hatchery. At hatching, 150 chicks of each breed were transferred to the Avian Conservation Centre of Local Genetic Resources of the University of Turin (Italy) (44°50′58″ N and 7°43′13″ E), which, in 2016, was recognized by the Italian Ministry of Agriculture and Forestry Policies. A vaccination program was applied against coccidiosis, Marek’s, and Newcastle diseases. In the Avian Conservation Centre, the unsexed chicks were weighed, labeled with a wing metal tag and reared in indoor pens (2.0 × 1.0 m) up to 8 w of age (25 birds/pen; 6 pens/breed). Birds were kept in a thermo-neutral zone and a 16L:8D (light:dark hours) lighting program was applied. At 8 w of age, the bird’s sex was identified by direct visual examination, the chicks were then separated by sex, selected on the base of average body weight (BW), and randomly transferred to growing pens (2.2 × 3.5 m) with access to an external paddock (2.2 × 4.5 m) (15 birds/pen; 4 pens/sex for each breed). At 8 w of age onwards, the natural photoperiod was applied (from June, 15L:9D, to November, 9L:15D). The BW was recorded biweekly for each bird from hatching (0 w) to 32 w of age, for a total of 17 weighings. The chickens always had free access to water and were fed ad libitum a standard commercial starter diet from hatching to 8 w of age (200 g/kg crude protein (CP), and 11.80 MJ/kg metabolizable energy (ME)), followed by a growing diet from 8 w to 32 w of age (185 g/kg CP and 12.20 MJ/kg ME).

### 2.3. Analysis of Growth Performance (Actual Data)

Growth analyses were carried out in R environment version 3.5.3 [29]. Analysis of variance (ANOVA) was performed to evaluate effects of some variables on the actual BW at different weeks of age using a linear model (LM) [30]:

*Y*i = *μ* + *Br*j + *Sx*k + *Br*j × *Sx*k + *Rl*m + *e* (LM1)

where *Y*i was BW of any chicken (dependent variable), *μ* was population mean, *Br*j was fixed effect of breed (j = 1 and 2), *Sx*k was fixed effect of sex (k = 1 and 2), (*Br*j × *Sx*k) was interaction in breed and sex combination, *Rl*m was random effect of relationship between chicks, and *e* was random residual error. In absence of any parentage information, molecular relationship was estimated as a proportion of shared alleles after microsatellite analysis [15]. Each independent variable in the model was tested with significance level *p* < 0.05 by backward elimination approach. Differences in average BW according to breed and sex were investigated by means of Waller–Duncan k-ratio *t*-test and significance was declared at *p* < 0.05.

### 2.4. Analysis of Growth Pattern According to the Gompertz Model (Estimated Data)

The Gompertz (G) model [27] (Equation (3)) was used to analyze the growth curve of the estimated BW of each chicken and to obtain the growth parameters (growth rate, inflection point, and adult BW) [31]:

BWt = BWa × exp (−*b* × exp(−*k*t)) (G1)

where BWt was the weight of any chicken at a given time t (w or d), BWa was upper asymptote or adult weight, *b* described the shape of growth curve being related to both BWa and weight at hatching or initial weight (BW0) (that is *b* = ln(BWa/BW0)), and *k* was instantaneous relative growth rate (d^−1^) affecting slope.

From the model, some parameters were derived having biological meaning because they were referred to the inflection point (ip), or time at which the growth rate reached its maximum. They were age Tip (w or d) and weight BWip (g), such as BWip = 0.368BWa (where 0.368 = 1/*e*, that is the constant of Gompertz model) and Tip = ln(*b*)/*k* [25,27]. The maximum growth rate (MGR) or rate at inflection point (g/d) was obtained as BWip × *k*. BWs was the estimated weight at the mean slaughter age or age for sale (180 d). A degree of maturity (Dm) was also computed as BWs/BWa.

A further Gompertz model was used [27] (Equation (7)), according to the following equation [32]:

BWt = BWa × exp {−exp ((*e* × *μ* / BWa) (*λ* − t) + 1)} (G2)

where BWt was weight of any chicken at time t, *λ* was lag time, that is the time (d) before the growth rate began to increase until its maximum was attained, and *μ* was absolute growth rate at inflection point (g/d); the components BWa and t were as for G1. Some parameters were derived from the model: Tip = t where BWt = BWip and BWs = BWt where t = 180 d. BWip and Dm were computed as for the model G1.

Both models G1 and G2 provided a curve of fitted BW with parameters for each chicken. Average values according to breed and sex were obtained for each parameter. ANOVA was performed using the model LM1 on the parameters as dependent variables. Differences between breed and sex were investigated by means of Waller–Duncan k-ratio *t*-test. Significance was declared at *p* < 0.05.

Correlation was estimated on the overall data set between (1) the estimated BW values obtained by the two models G1 and G2 and (2) the direct and derived parameters of each model.

Goodness of fit of the two models to the actual data was determined on each breed and sex separately using adjusted coefficient of determination (*r*^2^) and Akaike’s information criterion (AIC) as a residual error component.

### 2.5. Analysis of PAX7 Polymorphism and Its Association with Growth Parameters

In order to perform molecular analyses, blood samples were collected from 180 d old chickens by the wing vein in heparinized tube; a blood aliquot was immediately frozen at −20 °C pending DNA analysis. DNA was extracted with the NucleoSpin^®^ Blood QuickPure kit (Macherey-Nagel, Düren, Germany).

The PCR protocol of Zhang et al. [28] was used to amplify the *PAX7* fragment containing a 31bp indel that was reported to be a candidate marker in fast growing chickens. Explanation of the experimental details, namely sequencing of amplicons, aligning of sequences, designing a new primer pair, and genotyping by capillary electrophoresis was presented in Supplementary materials (Table S1) [33]. Expected genotypic frequencies under random mating and difference between breeds were evaluated using the FSTAT 2.9.3.2 software [34].

Analysis of association of body weight with effects of *PAX7* genotype at different weeks of age was performed on each breed–sex group using the linear model [30]:

*Y*i = *μ* + *G*j + *e* (LM2)

where *Y*i was BW of any chicken (dependent variable), *μ* was population mean, *G*j was fixed effect of *PAX7* genotype (j = 1 to 3), and *e* was the random residual error. ANOVA was performed using the model LM2 on the parameters as depend variables and differences between genotypes were investigated by means of Waller-Duncan k-ratio *t*-test. Significance was declared at *p* < 0.05.

## 3. Results

### 3.1. Analysis of Growth Performance (Actual Data)

For the purpose of the present trial, only birds with complete weight records (from week 0 up to 32 weeks of age) were considered, namely 46 BS females, 47 BS males, 54 BP females, and 54 BP males, for a total of 201 chickens. As a whole, the mortality rate was 3.75% and no significant difference were observed between breeds. Biweekly growth performances and results of the analysis of fixed effects (LM1) were reported in Table 1.

At hatching (w 0 = BW0), all BP and BS chicks weighed on average 39 g; thereafter, the growth rate became different between breeds being the BP heavier than the BS from 2 to 18 w of age. From 20 w of age onwards, the BW was no more influenced by breed. From 4 w of age onwards, the growth rate showed an increasing sexual dimorphism (*p* < 0.001) with males having higher average BW than females. At the standard slaughter age (26 w ≈ 180 d), males weighed more than females (about +33%).

The interaction breed–sex was not significant throughout the experimental period.

### 3.2. Analysis of Growth Pattern According to the Gompertz Model (Estimated Data)

The parameters of growth pattern of the G1 and G2 models were provided in Table 2. G1 showed a better fit than G2 due to a higher efficiency (*r*^2^) and a lower error component (AIC). Correlation between actual and estimated BW was also computed for each week of age on the overall sample (Figure S1); no correlation was detected at hatching, whereas the correlation between actual and estimated BW increased from week 2 (*p* < 0.001) and was higher in the G2 model up to 20 w of age.

**Figure 1:**
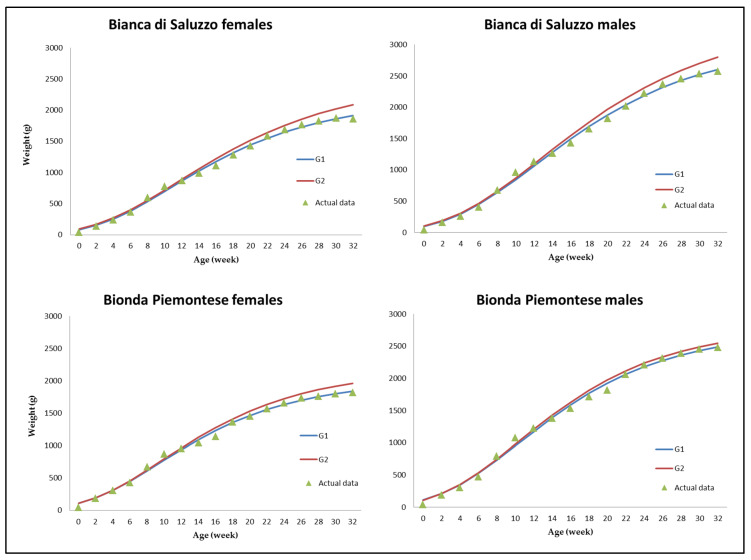
Predicted weight at different age for the *Bianca di Saluzzo* and *Bionda Piemontese* chicken breeds according to the Gompertz (G) model. Blue line, G1 model; red line, G2 model; triangle, actual data.

All the actual growth curves showed a biphasic trend between 13 and 21 w of age; as a consequence, before and after that period, the G1 and G2 models drew a regression curve that underfitted actual BW (Figure 1).

Correlation between direct and indirect parameters of each model was computed (Table S2). In general, the correlation between BWip and BWa was trivial because the first always derived from the latter by means of the constant 0.368 of the Gompertz model. In addition, the high correlation of BWa with Tip and BWs was expected because both parameters referred to the adult BW.

In the G1 model, parameter *b*, which was related to the shape of the growth curve, showed low positive correlation with BWa (Table S2) and was higher in BS than in BP and in males of both breeds (*p* < 0.001) (Table 2). The relative growth rate *k*, related to the slope of the curve, was higher in BP than in BS (*p* < 0.001) (Table 2). In this concern, a higher *k* value corresponded to a shorter time to reach maturity, namely, a shorter age at inflection point was related to a lower weight attained at that time and at the mature age (Table S2).

In the G2 model, the lag time *λ* showed moderate and positive correlation with all the other parameters (Table S2), namely the longer lag time (before the growth rate started to increase up to the inflection point), the higher values of mature weight and derived parameters were attained. The lag time was shorter in the BP than in the BS and in females of both breeds (*p* < 0.001) (Table 2).

The G1 and G2 models provided almost the same values of growth rate even if in G1 MGR was a derived parameter whereas in G2 *μ* was a direct parameter not affected by BWa [27] (Equation (7)). The correlation between weights at slaughter age, namely actual (w 26) and estimated (BWs), was +0.846 and +0.879 for G1 and G2, respectively (*p* < 0.001).

Both Gompertz models showed differences between breeds in all growth parameters except for BWs and MGR. In the two breeds, there was no correlation between *μ* and Tip (Table S2) and the same maximum growth rate was attained at different age. The BP reached Tip two weeks earlier than the BS (*p* < 0.001) (Table 2) with a lower BWip. Later on, at the average slaughter age, a lower Dm was observed in all BS chickens compared with BP chickens, even if BWs did not show difference between breeds (Table 2). At mature weight, BS was predicted to be heavier than BP (*p* < 0.001).

Estimated sex differences were observed in all parameters excluding the slope of growth curve (*k*) (Table 2). Females reached Tip ten days before males (*p* < 0.05), when they showed a lower BWip and a lower MGR (*p* < 0.001). The hens were not able to fill this gap, as it was observed on the actual data. Males were heavier than females (*p* < 0.001) at 180 d (BWs) and at mature age (BWa).

As previously stated on the observed data, no breed–sex interaction was detected using LM1 on the parameters of Gompertz models.

### 3.3. Comparison of Actual and Estimated Growth Data between the BS and BP and Other Chicken Breeds/Hybrids

The comparison of the actual growth phases of BP and BS with BW of different breeds were reported in Table 3 [35,36,37,38,39,40,41,42,43,44,45]. The two breeds ranked below broilers and heavy slow-growing breeds and above lightweight local breeds.

The growth parameters of the two breeds were compared with patterns of other breeds or hybrid lines that were analyzed using the model G1 or a derived version [27] (Equations (11) and (13)) (Table 4) [22,23,25,46]. In other investigations, the model directly provided Tip [27] (Equation (1)) (Table S3) [19,20,47,48,49].

When estimated BWip and BWa were applied as criteria, the BP and BS shared with other local breeds an intermediate position between broilers and lightest breeds. As regards Tip, in local breeds it ranged from 70 d to more than 100 d, unlike broilers, which concluded their productive cycle in less than 50 d. In the fast-growing broilers, the relative growth rate (*k*) was higher than in local breeds, whereas, within population, it usually exhibited a negative correlation with the other growth parameters (Table S2). The MGR was very different across breeds/hybrid lines ranging from more than 50 g/d in all fast-growing broilers to less than 10 g/d in the light breeds. In most of slow-growing breeds, MGR ranged from 10–15 (BS and BP) to 20–25 g/d.

As for other slow-growing breeds, males of BS and BP took more time to reach Tip (on average 12%) and achieved higher BWip (40%) than females. The sexual dimorphism at this stage was less marked in broilers, where males could also reach the maximum growth earlier than females (5%). In some local breeds, the relative growth rate (*k*), which affected the slope of growth curve, was lower in males than in females (10%–20%), according to the longer age at inflection point and the higher weights. BS and BP belonged to a group of slow-growing breeds with poor or no sexual dimorphism of *k* whereas, once again, the broilers differed because the males exhibited high relative growth rate.

### 3.4. Analysis of PAX7 Polymorphism and Its Association with Growth Parameters

*PAX7* amplicons showed polymorphic patterns on agarose gel electrophoresis in which two bands were detected of approximately 560 bp and 530 bp in size, respectively. The amplicons were then sequenced and the lengths of the two fragments were exactly 557 bp and 525 bp. The obtained sequences aligned with NC_006108.5 on exon 3 and intron 3 of the *PAX7* gene. The 557-bp amplicon included the sequence of the F allele reported by Zhang et al. [28], which was an intronic minisatellite region formed of two identical repeat units of 31 bp. The 525-bp amplicon contained only one unit with a single nucleotide deletion and corresponded to a new variant, which was called G allele (Figure S2).

By means of capillary electrophoresis, two alleles were found on the overall 201 chickens, F and G. There was no shift from the expected genotype frequencies under random mating (*p* > 0.05 after 2000 randomizations) (Table S4). The two breeds exhibited little differences in allele frequencies (*F*st = 0.028, *p* < 0.05); this meant that most genetic diversity depended on differences between individuals, included the chickens belonging to the same breed.

**Figure 2:**
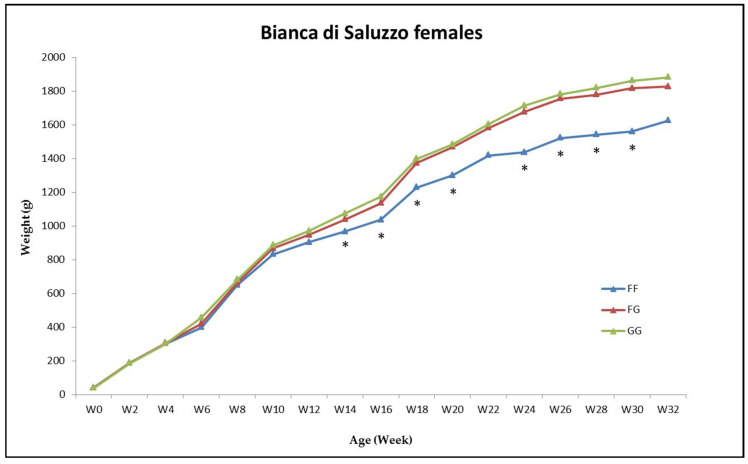
Average actual body weight (g) of the females of the *Bionda Piemontese* chickens during the growing phase divided into the three *PAX7* genotype groups *FF* (blue line), *FG* (red line) and *GG* (green line); * *p* < 0.05 among genotypes.

The two alleles showed association with the actual BW in the BP females starting from 14 w of age, where the FF hens differed from the GG hens (*p* < 0.05) (Figure 2) The Gompertz model G1 showed difference between genotypes concerning BWa and BWip; also BWs obtained using both G1 and G2 models was different (*p* < 0.05) (Table S5). The G allele always exhibited a more favorable effect than the F allele. The results of the other groups (BP males and BS of both sexes) showed no difference between genotypes (Tables S6–S8).

## 4. Discussion

The mathematical models have been applied mainly to the fast-growing chicken hybrids to predict the stage of maximum growth rate and the optimum age for slaughter and sale [21,26]. They can also be useful in (1) describing the resources of slow-growing breeds, (2) evaluating the ability of these breeds to adapt to particular rearing systems in a sustainable and profitable way, and (3) implementing the selection criteria, if necessary [22,23,24]. This investigation examines only the first feature, whereas the chickens have been managed according to very similar conditions of current farmer rearing systems.

In literature, different periods of age for weight measurements have been proposed to provide a reliable estimate of the parameters of a growth model, and they vary according to growth rate and management conditions of individual breeds and hybrid lines. The final age of observation is reported to be: 56–112 d for Ross broilers and Ross hybrids [19,25,46], 180 d for the Padovana Camosciata [22], 196 d for the junglefowl [49], and 240 d for Portuguese autochthonous breeds [48]. The present investigation includes 17 biweekly weighing per chicken (from w 0 to w 32) to perform the growth pattern analysis beyond the usual slaughter age, which is usually settled at 180 d for these slow-growing birds. To our knowledge, this is one of the longest experimental periods in this field, thus the estimations are expected to be reliable.

The Gompertz model is frequently used to study growth pattern and several versions exist [26,27]. The model G1 has been preferred here because it is frequently found in the literature on poultry science and provides parameters that are shared by other versions, so a comparison can be made with previous studies [19,20,22,23,25,29,46,47,48,49,50,51]. Moreover, correlations between G1 parameters may be confirmed [50]. An additional form of Gompertz equation was used, that is the model G2, which, as far as we know, has been applied to bacterial growth in food so far [27]. The G1 shows a better goodness of fit than G2 (*r*^2^, AIC) and, as a consequence, it provides an accurate estimate of BW during growth. The G2 provides the estimation of the time before the growth rate starts to increase towards its maximum, the so-called lag-time (*λ*), which is not included in the G1 model. In particular, *λ* highlights the differences of BW increase between breeds during the first stage of growing; to some extent (moderate correlation), the longer lag time, the higher Tip, BWip and BWa.

Both models provide an unreliable estimate of BW0, as expected [22,24,52]. However, in the present investigation the actual BW0 is known, whereas concordance with the actual BW rapidly increases from 2 w of age onwards.

The resulting growth pattern envisages that some chickens (1) belatedly show the rapid phase of growth, (2) belatedly attain the inflection point when they show a high weight, and (3) attain a high mature weight. The BS breed in comparison with the BP and the males of both breeds fit to this pattern. In particular, the maximum growth rate at inflection point shows an evident sexual dimorphism, being the males heavier than the females of both breeds. The two breeds exhibit the same growth rate, even if the BP attains this phase earlier at a lower BW.

Differences of the growth parameters demonstrate that the BS and BP are two populations with distinct characteristics. Furthermore, analysis of genetic structure and diversity using microsatellite markers infers that these breeds are separated from other Italian chicken breeds like the Livornese and Modenese and, at the same time, they are separated from each other, although they branch very closely in the cladogram depicting genetic distances between breeds [4].

In synthesis, if the two breeds are compared, the BS is more late-maturing and attains higher BW. The results on growth pattern show that the BS and BP are two distinct populations. Both breeds exhibit a growth sexual dimorphism from 4 w of age onwards.

With regards to the comparison with other slow-growing chicken breeds and commercial hybrids for the growth parameters of the Gompertz model, namely BWa, BWip, Tip, and MGR, the BP and BS breeds share with other slow-growing breeds—such as Padovana, Pedrês Portuguesa, and Preta Lusitânica [22,48]—an intermediate position between broilers, along with some medium or slow-growing hybrids, such as Hubbard and Berlanda [19,22,23,47], and the lightest chickens, such as the red junglefowl [48,49]. The BS is one of the most late-maturing populations having Tip of 86-96 d vs. 116–141 d of the White Plymouth Rock selected for low body weight [49]. Both Piedmontese breeds exhibit a slow growth rate, that is MGR 10–15 g/d vs. 20–25 g/d of other local breeds, compared to the lightest breeds as light White Plymouth Rock and red junglefowl which show < 10 g/d [49].

The sexual dimorphism distinguishes slow-growing chicken breeds from broiler chickens: the males of BS and BP exhibit high BWip and Tip, whereas males of broilers may reach the stage of maximum growth before the females, as it happens, for example, for the Ross 308 [19,46]. In general, males exhibit higher MGR than females.

Comparison of actual weights between different local poultry populations agrees with the estimated data in: (1) some heavy slow-growing chicken breeds, such as Robusta Maculata, Ermellinata di Rovigo and Milanino from the rural areas of Northern Italy [37,38,39,40], (2) a cluster of medium weight slow-growing chicken breeds, such as Castella Negra (Spain), BS, BP, and Padovana (Italy), and Bresse (France) [38,41,42,43,44], and (3) some other lightweight Italian chicken breeds, such as Modenese, Romagnolo, and Pepoi [38,45].

The actual growth curves show a biphasic trend at 13–21 w of age, which is before the chickens reach sexual maturity (20–24 w). A similar pattern has been described in the Mos breed (Galician) and Sasso T-44 line [53].

In synthesis, the growth parameters show that BS and BP are close to the unselected breeds. Among the Italian local poultry breeds, the BS and BP can be considered as slow-growing chickens with an intermediate size between heavy and light chicken breeds.

The chickens of the two breeds are usually slaughtered at 180 d, when only sexual dimorphism affects BW. In spite of this, the degree of maturity puts the BS at some disadvantage compared to the BP (Dm < 0.80) and its growing potential could be not fully exploited. Prediction of the best maturity for sale is a complex experimental task [54,55], particularly outside the industrial farming. Nevertheless, a rough estimate may be attempted. If a Dm ≥ 0.80 would be required, chickens should be reared above 180 d of age. However, postponing the age for sale could be economically questionable because of additional costs.

Selection to increased BW at different ages and improved performances has been widely evaluated in commercial lines and heritability of the growth curve parameters shows to be moderate to high [24,26,52,56]. A breeding strategy to modify the growth curve and, ultimately, Dm is feasible. In some slow-growing chickens, sex differences first occur from 14–21 d of age [23]. In chicks of BS and BP, the actual weights diverge after 2 w and 4 w between breeds and sexes, respectively; the lag time starts to highlight differences from 2 w of age onwards. It is worth investigating the real usefulness of this parameter as a selection criterion because the early growth may be useful in assessing the growth potential [56].

In local breeds, sustainability of a selection program must be carefully assessed [57]. Selection decreases effective population size, so a conservation project needs a precise strategy to restrain inbreeding, first [4,6]. Selection would emphasize sexual dimorphism in growth, feeding requirements and Dm at slaughter age because growth rate and Tip are controlled in part by different genes in males and females [52].

Selection could also modify the adaptation to low input rearing systems, which is the main conservation value of the local breeds and positively affects rearing performance. The BS and BP are well adapted to traditional conditions of rural areas and conserve ability to exploit free range [8,9]. As previously reported, in presence of novel stress and fear stimuli, such as crating associated with transport, the heterophil to lymphocyte ratio showed in the BS and BP resulted more constant than the Isa Brown strain [10].

As an alternative to selection and in absence of parentage information, a mating scheme based on molecular relationship is currently being carried out over some consecutive generations of the BP breed [16]. Investigation is still in progress, but the hypothesis is that the decrease of progeny inbreeding, in both females and males, could anticipate Tip and increase growth rate and BWip (Tip and MGR≈*μ* are not correlated). The Dm should increase with no additional costs. The mathematical models may also provide suitable information to optimize management (environmental conditions, diet) and evaluate effects on carcass components and meat quality to guarantee a source of niche high quality food [19,20,47,48,55,58].

In synthesis, improvement of performance at age for sale would be very profitable in slow-growing local breeds; nevertheless, an approach based on mating scheme to bring inbreeding under control could be the most proper way to attain this objective.

A detailed knowledge of genetic resources is useful to carry out precision livestock breeding and to estimate the conservation value of the local breeds [57,59,60]. Non-coding anonymous markers (microsatellite, SNP) are usually recommended for breed conservation purposes [61]. The molecular analysis using microsatellites shows that the chicken breeds of Piemonte retain a high level of genetic variation [4]. A particular contribution to conservation value derives also from candidate genes, which are genes exhibiting a function. Association studies need appropriate algorithms and a large well-planned sample that is hard to achieve from small local populations. Nevertheless, an exploratory investigation on the extant variation could be useful.

The *PAX7* gene plays a role in myogenesis of skeletal muscle and early development and its polymorphism is associated with growth performance in birds [62,63]. *PAX7* polymorphism in chicken is the result of a 31-bp indel into intron 3 [28]. The sequence alignment of the E and F alleles with the new G allele found in the BS and BP reveals the existence of a minisatellite region; tandem repetition of the 31-bp unit gives rise to the length variation based on three units (E allele), two units (F allele), and one unit (G allele).

In the present investigation, information on the hypothetical association of the F and G alleles with differences of growth patterns is restricted to the females of BP. Actual BW differences between genotypes start after 12 w of age. These results are consistent with Chang et al. [64], who reported that the expression of *PAX7* is lower during chicken embryo phase and it reaches the highest level in chicks at 8–10 w of age. The gene expression could be related, also, to the growth rate. In fact, the hybrid chickens reported by Zhang et al. [28] exhibit the association with BW from 4 w of age onwards—that is 8 w earlier than the BP. At 12 w of age, they weigh more than 1300 g, which is a BW that the BS and BP do not reach before 14–18 w. Consistent with this pattern, the estimated BW at 180 d of the BP hens shows association with the *PAX7* alleles, even if the Gompertz model exhibit poor differences. Zhang et al. [28] reported that the allele with the 31-bp deletion (F) is associated with disadvantage for growth, carcass, and meat quality traits, whereas a further deletion of allele G exhibits favorable association. These results and the intronic location of the minisatellite strengthen the hypothesis that E, F, and G alleles are just genetic markers.

As far as we know, this is the first contribution to the analysis of variation of the *PAX7* gene in a slow-growing breed. New alleles may be identified on the local breeds using candidate genes in order to have criteria of conservation value, though information on association with production traits is limited.

## 5. Conclusions

Two different forms of the Gompertz model provided useful information on growth pattern. Management system being equal, the model parameters show that the BS breed is more late-maturing (lag time, Tip, Dm) and attains higher BW (BWip, BWa) than the BP. In absence of any program of genetic improvement, the BS and BP are two populations with distinct characteristics.

Using BWa, BWip, Tip, and MGR as criteria, the comparison with several breeds/lines shows that the BS and BP are close to the unselected breeds. Among the Italian local poultry, they are slow growing, with an intermediate size between heavy and light chicken breeds.

In small size poultry population, a delicate balance between preservation of biodiversity and performance improvement should be considered. Improvement of performances would be very profitable in slow-growing chicken breeds; nevertheless, selection would increase inbreeding, emphasize growth sexual dimorphism, and modify the adaptation to low input environment, which is the main conservation value of the local breeds. An approach based on mating scheme to bring inbreeding under control, anticipate Tip, increase growth rate and BWip, and improve Dm is the most proper way to improve BP and BS breeding systems.

In local poultry breeds, association studies are hard to plan and information on linkage with production traits is limited. Nevertheless, to have additional criteria of conservation value, new alleles should be identified using genes known to be candidate for growth performances improvement.

## Figures and Tables

**Table 1:** Average actual body weight (g) of the males and females of the *Bianca di Saluzzo* and *Bionda Piemontese* chickens during the growing phase.

w	*Bianca di Saluzzo*		*Bionda Piemontese*		SEM	*p*		
	Females (n = 46)	Males (n = 47)	Females (n = 54)	Males (n = 54)		B	S	B × S
0	38.8	39.8	39.1	38.7	0.42	n.s.	n.s.	n.s.
2	138 ^a^	159 ^b^	186 ^c^	184 ^c^	7.5	***	n.s.	n.s.
4	235 ^a^	257 ^a^	304 ^b^	304 ^b^	9.2	***	n.s.	n.s.
6	365 ^a^	404 ^b^	429 ^b^	467 ^c^	10	***	***	n.s.
8	595 ^a^	674 ^b^	667 ^b^	787 ^c^	14	***	***	n.s.
10	772 ^a^	958 ^b^	868 ^c^	1076 ^d^	18	***	***	n.s.
12	868 ^a^	1132 ^b^	949 ^c^	1222 ^d^	18	***	***	n.s.
14	985 ^a^	1266 ^b^	1041 ^c^	1374 ^d^	20	***	***	n.s.
16	1103 ^a^	1430 ^b^	1135 ^a^	1532 ^c^	23	**	***	n.s.
18	1277 ^a^	1652 ^b^	1363 ^c^	1710 ^b^	28	*	***	n.s.
20	1425 ^a^	1820 ^b^	1452 ^a^	1812 ^b^	33	n.s.	***	n.s.
22	1582 ^a^	2019 ^b^	1568 ^a^	2060 ^b^	34	n.s.	***	n.s.
24	1684 ^a^	2219 ^b^	1658 ^a^	2208 ^b^	36	n.s.	***	n.s.
26	1766 ^a^	2367 ^b^	1733 ^a^	2307 ^b^	37	n.s.	***	n.s.
28	1825 ^a^	2451 ^b^	1761 ^a^	2384 ^b^	37	n.s.	***	n.s.
30	1868 ^a^	2536 ^b^	1798 ^a^	2450 ^b^	38	n.s.	***	n.s.
32	1854 ^a^	2572 ^b^	1819 ^a^	2475 ^b^	38	n.s.	***	n.s.

w: week of age at recording; n: number of birds. SEM: standard error of mean; values within a row with no common superscript letter (^a, b, c, d^) differ at *p* < 0.05 (Waller–Duncan test) between breed and sex. B, S, B × S: breed, sex and breed × sex interaction fixed effects on BW (model LM1); not significant (n.s.) *p* > 0.05, * *p* < 0.05, ** *p* < 0.01, *** *p* < 0.001.

**Table 2:** Growth curve parameters of the two forms of Gompertz model (G1 and G2) estimated on the males and females of *Bianca di Saluzzo* and *Bionda Piemontese* chicken breeds.

Item	*Bianca di Saluzzo*		*Bionda Piemontese*		SEM	*p*		
	Females (n = 46)	Males (n = 47)	Females (n = 54)	Males (n = 54)		B	S	B × S
***Model G1***								
BWa	2184 ^a^	3074 ^b^	201 ^c^	2745 ^d^	55	***	***	n.s.
*b*	3.32 ^a^	3.55 ^b^	2.99 ^c^	3.25 ^a^	0.05	***	***	n.s.
*k*	0.014 ^ab^	0.014 ^b^	0.016 ^c^	0.015 ^ac^	0.000	***	n.s.	n.s.
BWip	803 ^a^	1131 ^b^	740 ^c^	1010 ^d^	20	***	***	n.s.
Tip	86.0 ^a^	95.6 ^b^	71.6 ^c^	79.6 ^d^	1.9	***	***	n.s.
BWs	1647 ^a^	2188 ^b^	1634 ^a^	2177 ^b^	32	n.s.	***	n.s.
MGR	11.3 ^a^	15.0 ^b^	11.2 ^a^	15.0 ^b^	0.3	n.s.	***	n.s.
Dm	0.76 ^a^	0.72 ^b^	0.82 ^c^	0.79 ^c^	0.01	**	*	n.s.
*r*²	0.99	0.991	0.99	0.99	-	-	-	-
AIC	196	199	196	200	-	-	-	-
***Model G2***								
BWa	2508 ^a^	3383 ^b^	2193 ^c^	2819 ^d^	83	***	***	n.s.
*λ*	14.35 ^a^	20.63 ^b^	7.47 ^c^	12.58 ^a^	1.02	***	***	n.s.
*μ*	11.84 ^a^	15.83 ^b^	11.78 ^a^	15.65 ^b^	0.26	n.s.	***	n.s.
BWip	922 ^a^	1244 ^b^	807 ^c^	1037 ^d^	30	***	***	n.s.
Tip	94.1 ^a^	101.4 ^b^	77.5 ^c^	81.0 ^c^	2.4	***	*	n.s.
BWs	1748 ^a^	2302 ^b^	1718 ^a^	2225 ^b^	36	n.s.	***	n.s.
Dm	0.72 ^a^	0.69 ^a^	0.80 ^b^	0.79 ^b^	0.01	**	n.s.	n.s.
*r*²	0.97	0.99	0.98	0.99	-	-	-	-
AIC	206	201	197	200	-	-	-	-

SEM: standard error of mean; values within a row with no common superscript letter (^a, b, c, d^) differ at *p* < 0.05 (Waller–Duncan test) between breed and sex. Breed (B), Sex (S), and Breed × Sex (B × S): fixed effects on BW (model LM1); not significant (n.s.) *p* > 0.05, * *p* < 0.05, ** *p* < 0.01, *** *p* < 0.001. BWa: asymptotic body weight (g); *b*: shape parameter; *k*: coefficient of relative growth; Wip: body weight at inflection point (g); Tip: age at inflection point (d); BWs: body weight at the age of 180 d (g); MGR: maximum growth rate at inflection point (g/d); Dm (degree of maturity): BWs/BWa; *λ*: lag time (d); *μ*: absolute growth rate at inflection point (g/d); *r*^2^: (adjusted) goodness of fit criteria of the model; AIC: Akaike information criterion.

**Table 3:** Average actual body weight of the *Bianca di Saluzzo* and *Bionda Piemontese* chicken breeds during the growing phase: comparison with other breeds/hybrids.

Age (d)	RS	SA	RM	ER	MIL	CN	BS	BP	BR	PD	MO	RO	PE
42	2936					396	385	448					
84		2300				1095	1000	1086					
108									1300				
140				2186		1812	1623	1632	1800				
150					2381					1536			
168				2395									
175			2250										
180					2541		2068	2020		1800			
190				2900						2100			1500
210							2220	2147			2142	2175	

d: day. Values are average BW of males and females. RS: Ross 708 [35]; SA: SASSO [36]; RM: Robusta maculata [37]; ER: Ermellinata di Rovigo [37,38,39]; MIL: Milanino [40]; CN: Castellana Negra [41]; BS: Bianca di Saluzzo, present investigation; BP: Bionda Piemontese, present investigation; BR: Bresse [42]; PD: Padovana [38,43,44]; MO: Modenese [45]; RO: Romagnolo [45]; PE: Pepoi [38].

**Table 4:** Growth curve parameters according to the Gompertz model (G1) for the *Bianca di Saluzzo* and *Bionda Piemontese* chicken breeds: comparison with other breeds/hybrids. Data obtained from references.

**Item**	**RS1**			**RS2**			**HUB**			**B**		
	**F**	**M**	** Δ **	**F**	**M**	** Δ **	**F**	**M**	** Δ **	**F**	**M**	** Δ **
BWa	6401	6949	+9	4664	5475	+17	3657	4362	+19	2697	3880	+44
*b*	4.44	4.79	+8	4.20	4.62	+10	4.14	4.37	+6	4.03	4.39	+9
*k*	0.039	0.042	+8	0.036	0.036	0	0.031	0.031	0	0.021	0.019	−10
BWip	2356	2557		1716	2015		1345	1605		992	1427	
Tip	39	37	−5	43	43	0	46	48	+4	69	82	+19
MGR	92	107	+16	62	73	+18	42	50	+19	21	27	+29
**Item**	**BS**			**PA**			**BP**			**PC**		
	**F**	**M**	** Δ **	**F**	**M**	** Δ **	**F**	**M**	** Δ **	**F**	**M**	** Δ **
BWa	2184	3074	+41	2022	2245	+11	2012	2745	+36	1979	2558	+29
*b*	3.32	3.55	+6	4.35	4.77	+10	3.00	3.25	+10	4.01	4.16	+4
*k*	0.014	0.014	0	0.021	0.020	−5	0.016	0.015	−6	0.020	0.019	−5
BWip	804	1131		744	826		740	1010		728	941	
Tip	86	96	+12	75	81	+8	72	80	+11	72	76	+6
MGR	11	16	+45	16	17	+6	12	15	+25	15	18	+20

Data obtained from references where the model G1 or a derived version was used. F: females; M: males; Δ: ((male average − female average)/female average) × 100; BW*_a_*: asymptotic body weight (g); *b*: shape parameter; *k*: coefficient of relative growth; BWip: body weight at inflection point (g); Tip: age at inflection point (d); MGR: maximum growth rate at inflection point (g/d). Breed RS1: Ross 308 [46]; RS2: Ross 708 [25]; HUB: Hubbard, crossing JA57 × Redbro [23]; B: Berlanda [22]; BS: Bianca di Saluzzo, present investigation; PA: Padovana Argentata [22]; BP: Bionda Piemontese, present investigation; PC: Padovana Camosciata [22]. Except for [46] (which is a review), reported data do not consider any dietary treatment: for each trial, all birds received the same diet.

## Supplementary Materials

The following are available online at https://www.mdpi.com/2076-2615/10/6/969/s1, Figure S1: Correlation (*r*) between actual and estimated body weight using the G1 (solid line) and G2 (empty line) Gompertz models at different age for the *Bianca di Saluzzo* and *Bionda Piemontese* chicken breeds, Figure S2: Sequences of the *PAX7* alleles, Table S1: Genotyping for *PAX7* gene polymorphism by capillary electrophoresis, Table S2: Correlation between the direct and derived parameters of the two forms of Gompertz model (G1 and G2) estimated on the overall males and females of the *Bianca di Saluzzo* and *Bionda Piemontese* chicken breeds, Table S3: Gompertz model growth curve parameters [27] (Equation (1)) in other breeds/hybrids, Table S4. *PAX7* allele (*F* and *G*) and genotype (*FF*, *FG*, *GG*) frequencies in the *Bionda Piemontese* and *Bianca di Saluzzo* chicken breeds, Table S5: Average actual body weight (g) and growth curve parameters of the two forms of Gompertz model (G1 and G2) estimated on the females of *Bionda Piemontese* divided into the three *PAX7* genotype groups *FF*, *FG,* and *GG*, Table S6: Average actual body weight (g) and growth curve parameters of the two forms of Gompertz model (G1 and G2) of the males of *Bionda Piemontese* chickens during the growing phase divided into the three *PAX7* genotype groups *FF*, *FG,* and *GG*, Table S7: Average actual body weight (g) and growth curve parameters of the two forms of Gompertz model (G1 and G2) of the females of *Bianca di Saluzzo* chickens during the growing phase divided into the three *PAX7* genotype groups *FF*, *FG,* and *GG*, Table S8: Average actual body weight (g) and growth curve parameters of the two forms of Gompertz model (G1 and G2) of the males of *Bianca di Saluzzo* chickens during the growing phase divided into the three *PAX7* genotype groups *FF*, *FG,* and *GG*.

## References

[1] Scherf B.D., Pilling D., FAO (2015). The Second Reports of the State of the World’s Animal Genetic Resources for Food and Agriculture.

[2] Associazione Italiana Allevatori, Registro Anagrafico delle razze avicole autoctone. http://www.aia.it/aia-website/it/settori/area-tecnica/ufficio-sviluppo/registro-anagrafico-delle-razze-avicole-autoctone.

[3] Domestic Animal Diversity Information System. http://www.fao.org/dad-is/en/.

[4] Sartore S., Sacchi P., Soglia D., Maione S., Schiavone A., De Marco M., Ceccobelli S., Lasagna E., Rasero R. (2016). Genetic variability of two Italian indigenous chicken breeds inferred from microsatellite marker analysis. Brit. Poult. Sci..

[5] De Marco M., Dalmasso A., Bottero M.T., Pattono D., Sponza S., Sacchi P., Rasero R., Sartore S., Soglia D., Giacobini M. Local poultry breed assessment in Piemonte (northwest Italy). Proceedings of the 8th European Symposium on Poultry Genetics.

[6] Soglia D., Sacchi P., Sartore S., Maione S., Schiavone A., De Marco M., Bottero M.T., Dalmasso A., Pattono D., Rasero R. (2017). Distinguishing industrial meat from that of indigenous chickens with molecular markers. Poult. Sci..

[7] Ferrante V., Marelli S., Pignatelli P., Baroli D., Guidobono Cavalchini L. (2005). Perfomance and reactivity in three Italian chicken breeds for organic production. Anim. Sci. Pap. Rep..

[8] Schiavone A., Mellia E., Salamano G., Raccone V., Tarantola M., Nurisso S., Gennero S., Doglione L. (2009). Egg quality and blood parameters of “Bianca di Saluzzo” and Isa Brown hens kept under free range conditions. Ital. J. Anim. Sci..

[9] Ferrante V., Mugnai C., Ferrai L., Marelli S., Spagnoli E., Lolli S. (2016). Stress and reactivity in three Italian chicken breeds. Ital. J. Anim. Sci..

[10] De Marco M., Martinez Mirò S., Tarantola M., Bergagna S., Mallia E., Gennero M.S., Schiavone A. (2013). Effect of genotype and transport on tonic immobility and heterophil/lymphocyte ratio in two local Italian breeds and Isa Brown hens kept under free-range conditions. Ital. J. Anim. Sci..

[11] Strillacci M.G., Cozzi M.C., Gorla E., Mosca F., Schiavini F., Román-Ponce S.I., Ruiz López F.J., Schiavone A., Marzoni M., Cerolini S. (2017). Genomic and genetic variability of six Italian chicken populations using SNP and CNV as markers. Animal.

[12] Schiavone S., De Marco M., Dalmasso A., Bottero M.T., Pattono D., Sacchi P., Rasero R., Sartore S., Soglia D., Maione S. (2015). Preliminary Study on the carcass and meat characteristics of two free-range reared Italian localo hen breeds: Bianca di Saluzzo and Biona Piemontese. Ital. J. Anim. Sci..

[13] Mugnai C., Schiavone A., De Marco M., Sartore S., Soglia D., Maione S., Rasero R., Sacchi P., Dalmasso A., Bottero M.T. (2017). Market chain of “Consortium for safeguard of Bianca di Saluzzo e Bionda Piemontese breeds” in Piedmont: 2. Meat quality. Ital. J. Anim. Sci..

[14] Sartore S., Soglia D., Maione S., Dabbou S., Gariglio M., Sacchi P., Rasero R., Mugnai C., Gasco L., Gai F. (2019). Genetic diversity, productive and reproductive performance in Italian chicken breed Bianca di Saluzzo. Ital. J. Anim. Sci..

[15] Soglia D., Sartore S., Maione S., Gariglio M., Sacchi P., Rasero R., Mugnai C., Gasco L., Gai F., Schiavone A. (2019). Genetic diversity, productive and reproductive performance in Italian chicken breed Bionda Piemontese. Ital. J. Anim. Sci..

[16] Soglia D., Sartore S., Maione S., Dabbou S., Sacchi P., Rasero R., Mugnai C., Gasco L., Gai F., Schiavone S. (2019). Mating strategy based on DNA parentage information in Italian chicken breeds. Ital. J. Anim. Sci..

[17] Schiavone A., Brugiapaglia A., De Marco M., Sartore S., Soglia D., Maione S., Rasero R., Sacchi P., Dalmasso A., Bottero M.T. (2017). Market chain of “Consortium for safeguard of Bianca e Bionda breeds” in Piedmont: 1. demography, housing and slaughtering conditions. Ital. J. Anim. Sci..

[18] Conservation of Biodiversity in Italian Poultry Breeds (TuBAvI). https://www.pollitaliani.it/en/project/.

[19] Gous R.M., Moran E.T., Stilborn H.R., Bradford G.D., Emmans G.C. (1999). Evaluation of the parameters needed to describe the overall growth, the chemical growth, and the growth of feathers and breast muscles of broilers. Poult. Sci..

[20] Sakomura N.K., Longo F.A., Oviedo-Rondon E.O., Boa-Viagem C., Ferraudo A. (2005). Modeling energy utilization and growth parameter description for broiler chickens. Poult. Sci..

[21] Darmani-Kuhi H., Porter T., López S., Kebreab E., Strathe A.B., Dumas A., Dijkstra J., France J. (2010). A review of mathematical functions for the analysis of growth in poultry. Worlds Poult. Sci. J..

[22] Rizzi C., Contiero B., Cassandro M. (2013). Growth patterns of Italian local chicken populations. Poult. Sci..

[23] Narinç D., Aksoy T., Karaman E., Isaslan Curek D. (2010). Analysis of fitting growth models in medium growing chickens raised indoor system. Trends Anim. Vet. Sci. J..

[24] Barbato G.F. (1991). Genetic architecture of growth curve parameters in chckens. Theor Appl Genet..

[25] Darmani Kuhi H., Kebreab E., Lopez S., France J. (2003). An evaluation of different growth functions for describing the profile of live weight with time (age) in meat and egg strains of chicken. Poult. Sci..

[26] Narinç D., Narinç N.Ö., Aygün A. (2017). Growth curves analyses in poultry science. Worlds Poult. Sci. J..

[27] Tjørve K.M.C., Tjørve E. (2017). The use of Gompertz models in growth analyses, and new Gompertz-model approach: An addition to the unified-Richards family. PLoS ONE.

[28] Zhang S., Han R.L., Gao Z.Y., Zhu S.K., Tian Y.D., Sun G.R., Kang X.T. (2014). A novel 31-bp indel in the paired box 7 (*PAX7*) gene is associated with chicken performance traits. Brit. Poult. Sci..

[29] The R Project for Statistical Computing. https://www.r-project.org/.

[30] The R Project for Statistical Computing, package agricolae: Statistical Procedures for Agricultural Research. https://cran.r-project.org/web/packages/agricolae/agricolae.pdf.

[31] The R Project for Statistical Computing, Package Easynls: Easy Nonlinear Model. https://cran.r-project.org/web/packages/easynls/easynls.pdf.

[32] The R Project for Statistical Computing, Package Grofit: The Package was Developed to Fit many Growth Curves Obtained under Different Conditions. http://www2.uaem.mx/r-mirror/web/packages/grofit/grofit.pdf.

[33] Morgulis A., Coulouris G., Raytselis Y., Madden T.L., Agarwala R., Schäffer A.A. (2008). Database indexing for production MegaBLAST searches. Bioinformatics.

[34] Goudet J. FSTAT. https://www2.unil.ch/popgen/softwares/fstat.htm.

[35] Rogers A.G., Pritchett E.M., Alphin R.L., Brannick E.M., Benson E.R.I. (2015). Evaluation of the impact of alternative light technology on male broiler chicken growth, feed conversion, and allometric characteristics. Poult. Sci..

[36] Label Rouge: Pasture-Based Poultry Production in France. https://thepoultrysite.com/articles/label-rouge-pasturebased-poultry-production-in-france.

[37] Rizzi C., Chiericato G.M. (2010). Chemical composition of meat and egg yolk of hybrid and Italian breed hens reared using an organic production system. Poult. Sci..

[38] Zanetti E., De Morchi M., Dalvit C., Molette C., Remignon H., Cassandro M. (2010). Carcass characteristics and qualitative meat traits of three Italian local chicken breeds. Brit. Poult. Sci..

[39] Rizzi C., Baruchello M., Chiericato G.M. (2009). Effect of sex on slaughter performance and meat quality of Ermellinata di Rovigo chickens. Ital. J. Anim. Sci..

[40] Mosca F., Kuster C.A., Stella S., Farina G., Madeddu M., Zaniboni L., Cerolini S. (2016). Growth performance, carcass characteristics and meat composition of Milanino chickens fed on diets with different protein concentrations. Brit. Poult. Sci..

[41] Miguel J.A., Asenjo B., Ciria J., Calvo J.L. (2007). Growth and lay modelling in a population of Castellana Negra native Spanish hens. Brit. Poult. Sci..

[42] Verrier E., Tixier-Boichard M., Bernigaud R., Naves M. (2005). Conservation and value of local livestock breeds: Niche products and/or adaptation to specific environments. AGRI.

[43] Verdiglione R., Cassandro M. (2013). Characterization of muscle fiber type in the pectoralis major muscle of slow-growing local and commercial chicken strains. Poult. Sci..

[44] De Marchi M., Cassandro M., Lunardi E., Baldan G., Siegel P.B. (2005). Carcass characteristics and qualitative meat traits of the Padovana breed of chicken. Int. J. Poult. Sci..

[45] Sabbioni A., Zanon A., Beretti V., Superchi P., Zambini E.M. Carcass yield and meat quality parameters of two Italian autochthonous chicken breeds reared outdoor: Modenese and Romagnolo. Proceedings of the 12th European Poultry Conference.

[46] Demuner L.F., Suckeveris D., Muñoz J.A., Caetano V.C., Gonçalves de Lima C., Emygdio de Faria Filho D., Emygdio de Faria Filho D. (2017). Adjustment of growth models in broiler chickens. Pesq. Agropec. Bras..

[47] Michalczuk M., Damaziak K., Goryl A. (2016). Sigmoid models for the growth curves in medium-growing meat type chckens, raised under semi-confined conditions. Ann. Anim. Sci..

[48] Soares L.C., Lopes J.C., Brito N.V., Carvalheira J. (2015). Growth and carcass traits of three Portuguese autochthonous chicken breeds: Amarela, Preta Lusitânica and Pedrês Portuguesa. Ital. J. Anim. Sci..

[49] Sutherland D.-A.A.T., Ferst Honaker C., Dorshorst B., Andersson L., Brisbin I.L., Siegel P.B. (2018). Growth patterns for three generations of an intercross between red junglefowl and chickens selected for low body weight. J. Anim. Breed. Genet..

[50] Eleroğlu H., Yıldırım A., Şekeroğlu A., Ҫoksöyler F.N., Duman M. (2014). Comparison of growth curves by growth models in slow–growing chicken genotypes raised the organic system. Int. J. Agric. Biol..

[51] Masoudi A., Azarfar A. (2017). Comparison of nonlinear models describing growth curves of broiler chickens fed on different levels of corn bran. Int. J. Avian Wildlife Biol..

[52] Mignon-Grasteau S., Beaumont C., Le Bihan-Duval E., Poivey J.P., De Rochambeau H., Ricard F.H. (1999). Genetic parameters of growth curve parameters in male and female chcikens. Brit. Poult. Sci..

[53] Franco D., Rois D., Vázquez J.A., Lorenzo J.M. (2012). Comparison of growth performance, carcass components, and meat quality between Mos rooster (Galician indigenous breed) and Sasso T-44 line slaughtered at 10 months. Poult. Sci..

[54] Janisch S., Krischek C., Wicke M. (2011). Color values and other meat quality characteristics of breast muscles collected from 3 broiler genetic lines slaughtered at 2 ages. Poult. Sci..

[55] Dias R.C., Krabbe E.L., Bavaresco C., Stefanello T.B., Kawski V.L., Panisson J.C., Maiorka A., Roll V.F.B. (2020). Effect of strain and nutritional density of the diet on the water-protein ratio, fat and collagen levels in the breast and legs of broilers slaughtered at different ages. Poult. Sci..

[56] Aggrey S.E. (2002). Comparison of three nonlinear and spline regression models for describing chicken growth curves. Poult. Sci..

[57] FAO (2013). In Vivo Conservation of Animal Genetic Resources.

[58] Caldas J.V., Boonsinchai N., Wang J., England J.A., Coon C.N. (2019). The dynamics of body composition and body energy content in broilers. Poult. Sci..

[59] Flint A.P.F., Woolliams J.A. (2008). Precision animal breeding. Phil. Trans. R Soc. B..

[60] Georges M., Charlier C., Hayes B. (2019). Harnessing genomic information for livestock improvement. Nat. Rev. Genet..

[61] FAO (2011). Molecular Genetic Characterization of Animal Genetic Resources.

[62] Halevy O., Piestun Y., Allouh M.Z., Rosser B.W.C., Rinkevich Y., Reshef R., Rozenboim I., Wleklinski-Lee M., Yablonka-Reuveni Z. (2004). Pattern of *Pax7* expression during myogenesis in the posthatch chicken establishes a model for satellite cell differentiation and renewal. Dev. Dynam..

[63] Wang C., Yang Y.Z., Liu Y., Gong S.M., Wang H.Y., He D.Q. (2017). Paired box 7 (*Pax7*) gene: Molecular characterisation, polymorphism and its association with growth performance in goose (Anser cygnoides). Brit. Poult. Sci..

[64] Chang G.B., Liu X.P., Liao J., Chen R., Luan D.Q., Zhang Y., Dai A.Q., Ma T., Zhou W., Wang K.H. (2011). Temporal and spatial expression of the Pax-7 gene during chicken embryo and postnatal development. J. Anim. Vet. Adv..

