# Supplementary material for: Growth Performance Analysis of Two Italian Slow-Growing Chicken Breeds: Bianca di Saluzzo and Bionda Piemontese
Source: Animals (Basel). 2020 Jun 3;10(6):969. doi: 10.3390/ani10060969 (PMC7341298; doi:10.3390/ani10060969)
Supplement: Supplementary file 1 [file animals-10-00969-s001.pdf]

Table S1. Genotyping for *PAX7* gene polymorphism by capillary electrophoresis.

PCR conditions		
Forward primer: 5′-CTTGCTCCTTGCTCTGCATG-3′ (6Fam labelled)<br>Reverse primer: 5′-CAGACCCTCAGCACAACTCA-3′		
PCR mix (10 μl)	DNA	50 ng
	dNTPs	0.50 mM
	each primer	0.2 mM
	HotStarTaq® DNA Polymerase[1]	0.75 U
	95° for 10 min	
30 cycles	95° for 1 min	
	58° for 30 sec	
	72° for 30 sec	
Final extension	72° for 10 min	
**Capillary electrophoresis equipment[2]**		
Amplicon separation	ABI PRISM® 310 Genetic Analyzer	
Allele sizing	GeneScan™ 500 LIZ™ dye Size Standard	
Genotype calling	GeneMapper4.0 software	

[1]Qiagen, Hilden, Germany. [2]ThermoFisher Scientific, Waltham, MA, USA.

On a subsample of 20 chickens, the PCR protocol of Zhang et al. [28] was used to amplify the *PAX7* fragment containing a polymorphic sequence which was reported to be a candidate marker in fast growing chickens. The amplicons were separated on 2.5% agarose gel and sequenced with the PCR primers using the BigDye Terminator v1.1 Cycle Sequencing Kit (ThermoFisher Scientific, Waltham, MA, USA). Sequencing was carried out on an ABI PRISM® 310 Genetic Analyzer (ThermoFisher Scientific, Waltham, MA, USA). The amplicon sequences were aligned against public databases using the MegaBlast platform [33]. The DNA region containing the polymorpohic sequence was identified on the reference sequence NC_006108.5 (*PAX7* gene was annotated at position 4441852-4530024) and its structure was investigated. On the NC_006108 reference sequence, a new forward primer was designed near to the polymorphic sequence to obtain amplicons easy to separate by means of capillary electrophoresis. The new primer pair, PCR conditions, and capillary electrophoresis are described in Table S1 and was used to genotype all the chickens.

Table S2. Correlation between the direct and derived parameters of the two forms of Gompertz model (G1 and G2) estimated on the overall males and females of the *Bianca di Saluzzo* and *Bionda Piemontese* chicken. breeds.

Model G1			
**Item**	**BWa**	***b***	***k***
b	+0.318 ***		
k	–0.527 ***	+0.306 ***	
**BWip**	(+1)	+0.318 ***	–0.527 ***
**Tip**	+0.709 ***	+0.246 ***	–0.812 ***
**BWs**	+0.829 ***	+0.417 ***	–0.071 n.s.

Model G2			
**Item**	**BWa**	λ	μ
λ	+0.663 ***		
μ	+0.619 ***	+0.523 ***	
**BWip**	(+1)	+0.663 ***	+0.619 ***
**Tip**	+0.808 ***	+0.681 ***	+0.113 n.s.
**BWs**	+0.791 ***	+0.462 ***	+0.921 ***

not significant (n.s.) $p > 0.05$, * $p < 0.05$, ** $p < 0.01$, *** $p < 0.001$.

Table S3. Growth curve parameters (Gompertz model, equation 1) in other breeds/hybrids. Data obtained from references [27].

(b)	CR			RS3			CS			GP			PP			PL			AM			LWS			RJF		
	**F**	**M**	***Δ***	**F**	**M**	***Δ***	**F**	**M**	***Δ***	**F**	**M**	***Δ***	**F**	**M**	***Δ***	**F**	**M**	***Δ***	**F**	**M**	***Δ***	**F**	**M**	***Δ***	**F**	**M**	***Δ***
**BWa**	5217	6087	*+16*	4801	6050	*+26*	4085	5888	*+44*	3964	5922	*+49*	2344	3244	*+38*	2148	3048	*+42*	1952	2851	*+46*	957	1684	*+76*	568	763	*+34*
k	0.036	0.038	*+6*	0.036	0.039	*+8*	0.037	0.038	*+3*	0.030	0.030	*0*	0.019	0.015	*–21*	0.022	0.019	*–14*	0.025	0.022	*–12*	0.014	0.012	*–14*	0.027	0.023	*–15*
**BW_{ip}**	1920	2240		1767	2226		1503	2167		1459	2179		862	1193		790	1121		718	1049		352	620		209	281	
**T_{ip}**	45	43	*–4*	38	39	*+3*	43	44	*+2*	41	47	*+15*	74	82	*+11*	72	79	*+10*	69	77	*+12*	116	141	*+22*	56	65	*+16*
**MGR**	69	85	*+23*	64	87	*+36*	56	82	*+46*	44	65	*+48*	16	18	*+13*	17	21	*+24*	18	23	*+28*	5	7	*+40*	6	7	*+17*

F: females; **M**: males; ***Δ***: ((male average – female average)/female average) × 100; **BW_a**: asymptotic body weight (g); ***k*:** coefficient of relative growth; **BWip:** body weight at inflection point (g); **Tip**: age at inflection point (d); **MGR**: maximum growth rate at inflection point (g/d);. **Breed** CR: crossing Ross × Arbor Acres [19]; RS3: unspecified Ross line [20]; CS: crossing Steggles × Arbor Acres [19]; GP: crossing Polish Greenleg Partridge × fast-growing commercial broiler [47]; PP: Pedrês Portuguesa [48]; PL: Preta Lusitânica [48]; AM: Amarela [48]; LWS: White Plymouth Rock selected for low body weight at 56 d [49]; RJF: red junglefowl [49].

Table S4. *PAX7* allele (*F* and *G*) and genotype (*FF*, *FG*, *GG*) frequencies in the *Bionda Piemontese* and *Bianca di Saluzzo* chicken breeds.

Breed and gender	*F*	*G*	*FF*	*FG*	*GG*	**Ho**	**He**	
**Bianca di Saluzzo**	0.53	0.47	0.28	0.47	0.25	0.46	0.49	n.s.
Females	0.55	0.45	0.32	0.48	0.20	0.50	0.49	n.s.
Males	0.49	0.51	0.27	0.46	0.27	0.43	0.50	n.s.
**Bionda Piemontese**	0.40	0.60	0.17	0.47	0.36	0.47	0.48	n.s.
Females	0.40	0.60	0.13	0.54	0.33	0.54	0.48	n.s.
Males	0.41	0.59	0.20	0.41	0.39	0.41	0.48	n.s.

Ho: observed heterozygosity. **He:** expected heterozygosity under random mating. n.s.: not significant departure of Ho from He ($p > 0.05$ after 2000 randomisations).

Table S5. Average actual body weight (g) and Growth curve parameters of the two forms of Gompertz model (G1 and G2) estimated on the females of *Bionda Piemontese* divided into the three *PAX7* genotype groups *FF*, *FG* and *GG*.

**w**	**Bionda Piemontese Females**			SEM	*p*
	FF (n = 7)	***FG*** (n = 29)	***GG*** (n = 18)		
**0**	39.6	39.2	38.7	0.7	n.s.
**2**	186	186	185	15	n.s.
**4**	302	305	302	18	n.s.
**6**	399	420	456	17	n.s.
**8**	650	662	681	19	n.s.
**10**	831	866	885	23	n.s.
**12**	903	946	970	22	n.s.
**14**	966 ^{a}	1039 ^{ab}	1073 ^{b}	24	*
**16**	1037 ^{a}	1135 ^{ab}	1173 ^{b}	27	*
**18**	1228 ^{a}	1373 ^{ab}	1399 ^{b}	41	*
**20**	1300 ^{a}	1469 ^{b}	1484 ^{b}	43	*
**22**	1419	1581	1604	51	n.s.
**24**	1437 ^{a}	1677 ^{b}	1714 ^{b}	56	*
**26**	1522 ^{a}	1754 ^{b}	1781 ^{b}	58	*
**28**	1541 ^{a}	1778 ^{b}	1818 ^{b}	60	*
**30**	1559 ^{a}	1816 ^{b}	1862 ^{b}	65	*
**32**	1625 ^{a}	1827 ^{ab}	1881 ^{b}	66	n.s.
Item	**Model G1**				
**BWa**	1721 ^{a}	2030 ^{ab}	2094 ^{b}	92	*
b	2.82	3.01	3.02	0.09	n.s.
k	0.016	0.015	0.015	0.001	n.s.
**BWip**	633 ^{a}	746 ^{ab}	770 ^{b}	34	*
**Tip**	64.5	72.2	73.5	2.7	n.s.
**BWs**	1449 ^{a}	1648 ^{b}	1683 ^{b}	50	*
**MGR**	10.1	11.3	11.5	0.3	n.s.
**Dm**	0.85	0.82	0.81	0.02	n.s.
Item	**Model G2**				
**BWa**	1809	2273	2212	108	n.s.
λ	3.9	8.6	7.1	1.2	n.s.
μ	10.86	11.85	12.03	0.34	n.s.
**BWip**	665	836	813	40	n.s.
**Tip**	67.0	80.4	76.8	3.1	n.s.
**BWs**	1524 ^{a}	1734 ^{b}	1766 ^{b}	53	*
**Dm**	0.85	0.79	0.80	0.20	n.s.

Values are average on overall chickens; **w**: week of age; **n**: number of birds; **common superscript letter**= Waller-Duncan test ($p < 0.05$) between genotypes; **SEM**= standard error of mean; ***p*** = p-value ANOVA test: not significant (n.s.) $p > 0.05$, *$p < 0.05$. For both models, the direct parameters are in the top of the table, the derived parameters are in the bottom. **BWa:** asymptotic body weight (g); ***b***: shape parameter; ***k*:** coefficient of relative growth; **BWip**: body weight at inflection point (g); **Tip:** age at inflection point (d); **BWs**: body weight at the age of 180 d (g); **MGR**: maximum growth rate at inflection point (g/d); **Dm** (degree of maturity): BWs/BWa; λ**:** lag time (d); μ**:** absolute growth rate at inflection point (g/d).

Table S6. Average actual body weight (g) and growth curve parameters of the two forms of Gompertz model (G1 and G2) of the males of *Bionda Piemontese* chickens during the growing phase divided into the three *PAX7* genotype groups *FF*, *FG* and *GG*.

**w**	**Bionda Piemontese Males**			SEM	*p*
	FF (n = 11)	***FG*** (n = 22)	***GG*** (n = 21)		
**0**	38.71	39.07	38.28	0.72	n.s.
**2**	212	172	183	11	n.s.
**4**	334	290	301	13	n.s.
**6**	464	475	461	18	n.s.
**8**	791	798	773	27	n.s.
**10**	1079	1097	1052	35	n.s.
**12**	1219	1239	1207	39	n.s.
**14**	1352	1395	1362	43	n.s.
**16**	1501	1544	1536	48	n.s.
**18**	1690	1714	1718	56	n.s.
**20**	1865	1803	1794	69	n.s.
**22**	2078	2057	2053	75	n.s.
**24**	2222	2200	2209	81	n.s.
**26**	2320	2287	2322	77	n.s.
**28**	2396	2375	2388	75	n.s.
**30**	2469	2428	2464	75	n.s.
**32**	2512	2456	2476	76	n.s.
Item		**Model G1**			
**BWa**	2824	2692	2760	92	n.s.
b	3.16	3.28	3.27	0.05	n.s.
k	0.014	0.015	0.015	0.000	n.s.
**BWip**	1039	990	1015	33	n.s.
**Tip**	81.9	77.5	80.7	2.1	n.s.
**BWs**	2187	2170	2177	69	n.s.
**MGR**	14.67	15.25	14.92	0.57	n.s.
**Dm**	0.78	0.81	0.79	0.01	n.s.
Item		**Model G2**			
**BWa**	2981	2726	2831	75	n.s.
λ	12.8	12.1	13.0	1.3	n.s.
μ	15.6	15.9	15.4	0.5	n.s.
**BWip**	1097	1003	1041	1.0	n.s.
**Tip**	84.6	77.7	82.6	48	n.s.
**BWs**	2271	2203	2225	3	n.s.
**Dm**	0.75	0.82	0.79	0.02	n.s.

Values are average on overall chickens; **w**: week of age; **n**: number of birds; **SEM**= standard error of mean; ***p*** = p-value ANOVA test: n.s., not significant.

For both models, the direct parameters are in the top of the table, the derived parameters are in the bottom.

BWa: asymptotic body weight (g); ***b***: shape parameter; ***k*:** coefficient of relative growth; **BWip**: body weight at inflection point (g); **Tip**: age at inflection point (d); **BWs**: body weight at the age of 180 d (g); **MGR**: maximum growth rate at inflection point (g/d); **Dm** (degree of maturity): BWs/BWa; λ**:** lag time (d); μ**:** absolute growth rate at inflection point (g/d).

Table S7. Average actual body weight (g) and growth curve parameters of the two forms of Gompertz model (G1 and G2) of the females of *Bianca di Saluzzo* chickens during the growing phase divided into the three *PAX7* genotype groups *FF*, *FG* and *GG*.

w	**Bianca di Saluzzo Females**			SEM	*p*
	FF (n = 14)	*FG* (n = 23)	*GG* (n = 9)		
**0**	38.33	38.37	41.09	0.88	n.s.
**2**	129	141	153	10	n.s.
**4**	236	239	234	13	n.s.
**6**	385	367	339	16	n.s.
**8**	633	587	583	24	n.s.
**10**	822	761	766	35	n.s.
**12**	905	857	885	38	n.s.
**14**	1020	976	1000	39	n.s.
**16**	1134	1093	1124	44	n.s.
**18**	1319	1266	1293	52	n.s.
**20**	1470	1415	1448	61	n.s.
**22**	1634	1560	1626	68	n.s.
**24**	1737	1669	1708	74	n.s.
**26**	1783	1754	1807	72	n.s.
**28**	1835	1820	1853	68	n.s.
**30**	1850	1878	1904	71	n.s.
**32**	1883	1838	1886	73	n.s.
Item		**Model G1**			
**BWa**	2222	2219	2035	88	n.s.
b	3.33	3.34	3.26	0.10	n.s.
k	0.014	0.015	0.014	0.000	n.s.
**BWip**	817	816	749	32	n.s.
**Tip**	85.9	86.1	85.8	4.0	n.s.
**BWs**	1676	1675	1527	52	n.s.
**MGR**	11.5	11.6	10.4	0.5	n.s.
**Dm**	0.76	0.77	0.76	0.02	n.s.
Item		**Model G2**			
**BWa**	2317	2581	2616	214	n.s.
λ	12.2	15.7	14.2	2.5	n.s.
μ	11.2	12.1	12.1	0.6	n.s.
**BWip**	852.5	949.6	962.3	78.9	n.s.
**Tip**	89.7	96.0	96.2	6.7	n.s.
**BWs**	1684	1793	1734	69	n.s.
**Dm**	0.7	0.7	0.7	0.03	n.s.

Values are average on overall chickens; **w**: week of age; **n**: number of birds; **SEM**= standard error of mean; ***p*** = p-value ANOVA test: n.s., not significant. For both models, the direct parameters are in the top of the table, the derived parameters are in the bottom. **BWa:** asymptotic body weight (g); ***b***: shape parameter; ***k*:** coefficient of relative growth; **BWip**: body weight at inflection point (g); **Tip**: age at inflection point (d); **BWs**: body weight at the age of 180 d (g); **MGR**: maximum growth rate at inflection point (g/d); **Dm** (degree of maturity): BWs/BWa; λ: lag time (d); μ: absolute growth rate at inflection point (g/d).

Table S8. Average actual ***body*** weight (g) and growth curve parameters of the two forms of Gompertz model (G1 and G2) of the males of *Bianca di Saluzzo* chickens during the growing phase divided into the three *PAX7* genotype groups *FF*, *FG* and *GG*.

w	**Bianca di Saluzzo Males**			SEM	*p*
	FF (n = 14)	***FG*** (n = 20)	***GG*** (n = 13)		
**0**	39.8	40.3	38.8	4.0	n.s.
**2**	170	146	162	26	n.s.
**4**	250	251	267	37	n.s.
**6**	402	378	436	55	n.s.
**8**	676	647	692	82	n.s.
**10**	954	925	979	111	n.s.
**12**	1122	1095	1166	129	n.s.
**14**	1257	1240	1282	140	n.s.
**16**	1415	1415	1440	158	n.s.
**18**	1626	1653	1642	182	n.s.
**20**	1797	1801	1827	210	n.s.
**22**	2003	2037	1961	217	n.s.
**24**	2192	2229	2187	238	n.s.
**26**	2363	2360	2365	255	n.s.
**28**	2455	2421	2469	267	n.s.
**30**	2564	2505	2532	271	n.s.
**32**	2608	2548	2548	275	n.s.
Item		**Model G1**			
**BWa**	3158	3038	3039	361	n.s.
b	3.64	3.39	3.68	0.4	n.s.
k	0.014	0.013	0.014	0.002	n.s.
**BWip**	1161	1117	1118	133	n.s.
**Tip**	96	95	95	12	n.s.
**BWs**	2239	2144	2202	233	n.s.
**MGR**	15.6	14.4	15.4	1.7	n.s.
**Dm**	0.73	0.72	0.73	0.08	n.s.
Item		**Model G2**			
**BWa**	3320	3315	3557	418	n.s.
λ	20.9	18.5	23.7	3.7	n.s.
μ	16.2	15.2	16.1	1.8	n.s.
**BWip**	1221	1220	1308	154	n.s.
**Tip**	98	100	107	13	n.s.
**BWs**	2326	2272	2323	248	n.s.
**Dm**	0.71	0.70	0.67	0.08	n.s.

Values are average on overall chickens; **w**: week of age; **n**: number of birds; **SEM**= standard error of mean; ***p*** = p-value ANOVA test: n.s., not significant. For both models, the direct parameters are in the top of the table, the derived parameters are in the bottom. **BWa:** asymptotic body weight (g); ***b***: shape parameter; ***k*:** coefficient of relative growth; **BWip**: body weight at inflection point (g); **Tip**: age at inflection point (d); **BWs**: body weight at the age of 180 d (g); **MGR**: maximum growth rate at inflection point (g/d); **Dm** (degree of maturity): BWs/BWa; λ**:** lag time (d); μ**:** absolute growth rate at inflection point (g/d).

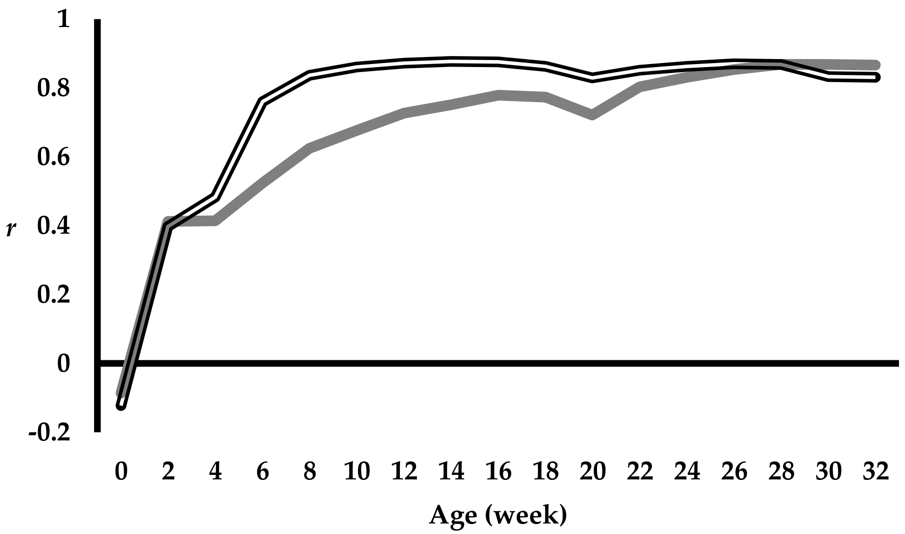

Figure S1. Correlation (*r*) between actual and estimated body weight using the G1 (solid line) and G2 (empty line) Gompertz models at different age for the *Bianca di Saluzzo* and *Bionda Piemontese* chicken breeds. Values were obtained for each wk on the overall sample.

Allele E

GGTAGACCCCAAAGTAGGGTCGAGGGGACGTCAGCCCATGGAAAGTAGGGTCGAGGGGACGTCAGCCCATGGAAAGTAGGGTCGAGGGGACGTCAGCCCATGGCTTGCTCACT

Allele F GGTAGACCCC**AAAGTAGGGTCGAGGGGACGTCAGCCCATGGAAAGTAGGGTCGAGGGGACGTCAGCCCATGG**-------------------------------CTTGCTCACT

Allele G GGTAGACCCC**AAAGTAGGGTCGAGGGGACG-CAGCCCATGG**--CTTGCTCACT

Figure S2. Sequences of the *PAX7* alleles. Allele E was found by Zhang et al. [28]. Allele F was found by Zhang et al. [28] and in the present investigation. Allele G contained a T deletion and was found in the present investigation only. The sequences were aligned on the GenBank sequence NC_006108.5.
